# Effects of Environmental Organic Pollutants on Environment and Human Health: The Latest Updates

**DOI:** 10.3390/toxics12040231

**Published:** 2024-03-22

**Authors:** Zhen-Guang Yan, Zhi-Gang Li, Jin-Zhe Du

**Affiliations:** 1 State Key Laboratory of Environmental Criteria and Risk Assessment, Chinese Research Academy of Environmental Sciences, Beijing 100012, China; lizg@craes.org.cn; 2 College of Marine Science and Technology, Qingdao Agricultural University, Qingdao 266109, China; djz@qau.edu.cn

## 1. Introduction

This editorial introduces the Special Issue “Effects of Environmental Organic Pollutants on Environment and Human Health: The Latest Updates”. Environmental Organic Pollutants include volatile organic compounds (VOCs) and persistent organic pollutants (POPs). VOCs originate from motor vehicle emissions and various manufactured products, such as building materials, paints, and cleaning agents, which often pollute the atmosphere around us. POPs have an intrinsic resistance to natural degradation processes and are found in polluted water, soil, atmosphere, sediment, etc. In addition, many emerging organic pollutants are considered potentially harmful to human health [1], such as Pharmaceuticals and Personal Care Products, and thus they are prioritized in biomonitoring surveillance.

This Special Issue presents the most recent advancements in research on VOCs and POPs, encompassing investigations and risk assessments of environmental organic pollutants, studies on the toxic mechanisms of such pollutants, as well as comprehensive summaries and reviews of the research progress and historical developments within related fields.

## 2. An Overview of Published Articles

There are two articles focused on VOCs. Wang et al.’s (contribution 4) field observations of CO, NO, NO_2_, O_3_, and VOCs were conducted in Beijing, Baoding, and Shanghai. They illustrated the pollution characteristics, source analysis, and risk assessment of VOC in the three cities. The research results indicated that motor vehicle exhaust was the main source of VOCs in all three cities. Acrolein was the only substance with an average hazard quotient greater than 1, indicating a significant non-carcinogenic risk. In Beijing, 1,2-dibromoethane had an R-value of 1.1 × 10^−4^ and posed a definite carcinogenic risk. Singh et al. (contribution 10) used statistical analysis to determine that anthropogenic activities were considerable sources of emission of VOCs in industrial areas. During the lockdown, the major factors behind the crucial decrease in TVOC levels were the complete and partial restrictions on industrial activities, transport, and marketplace openings. Comparatively, the lifetime cancer risk (LCR) value for males and females was estimated to be higher throughout the lockdown period than in the pre-and post-lockdown periods. These findings showed that exposure to VOCs induced adverse health effects, including carcinogenic and non-carcinogenic risks.

There are 11 articles focused on POPs. The research fields of these studies include toxic mechanisms, epidemiology, and ecological risk. 

Among them, there are two articles on the toxic mechanisms of exposure to POPs on health. Exposure to Bisphenol A (BPA) has led to an increased risk of obesity and nonalcoholic fatty liver diseases (NAFLDs). Li et al. (contribution 2) investigated the effects of BPA on the hepatic lipid metabolism function and its potential mechanisms in mice through a comparison of the BPA exposure model and the BPA exposure + cessation of drug treatment model. The results showed that the mice exposed to BPA manifested NAFLD features. Importantly, BPA could significantly decrease the level of APOD protein, whereas an extremely significant increase occurred after they stopped exposure. Meanwhile, APOD over-expression suppressed TG accumulation in AML12 cells. In conclusion, the damage caused by BPA can be repaired by upregulation of APOD, and it is a potentially effective biochemical detection indicator for the treatment of obesity or NAFLDs caused by BPA exposure. The persistent pollutants in wastewater can enter the food chain and ultimately endanger human health [2]. Yang et al. (contribution 3) studied the cytotoxicity of the industrial wastewater treatment. They conducted a broad evaluation of the environmental health risks from industrial wastewater along the Yangtze River, China, using a battery of bioassays. The toxicity tests on the wastewater samples showed that the wastewater treatment processes were effective at lowering acetylcholinesterase (AChE) inhibition, HepG2 cells’ cytotoxicity, the estrogenic effect in T47D-Kbluc cells, DNA damage in Euglena gracilis, and the mutagenicity of *Salmonella typhimurium*. These two studies provide a good basis for the health risk assessment of BPA and industrial wastewater, and Yang et al.’s finding also provides a scientific reference for the optimization and operation of wastewater treatment processes. 

Furthermore, in this Special Issue, Niu et al. (contribution 11) systematically summarize contemporary findings from epidemiological surveys, and they explored the mechanistic correlation between exposure to emerging pollutants (including endocrine disruptors, perfluorinated compounds, microplastics, and antibiotics) and blood glucose dysregulation. Their work provides a basic reference for further research on the complex interaction between new pollutants and diabetes.

It is clear that a poor ecological environment will damage our health. Ecological risks are closely related to health risks. In this Special Issue, there are eight articles that focus on the ecological risk of organic matter. Wang et al. (contribution 1) researched the nine pesticide pollutants included in the “List of New Key Pollutants for Control (2023 Edition)” issued by the Chinese government. They analyzed the environmental exposure to pesticide pollutants in sediments along the coast of China and derived baseline standards for sediment quality using the balanced distribution method. They also conducted a multi-level ecological risk assessment of pesticides in sediment. The risk quotient assessment showed that endosulfan and DDT posed medium environmental risks to the Chinese coastal sediment environment, and PCBs posed medium risks in some bays of the East China Sea. The semi-probabilistic optimized evaluation and the joint probability curve (JPC) assessments all showed that endosulfan and DDT pose a certain degree of risk to the environment. Hong et al. (contribution 5) analyzed the spatial distribution characteristics of nine alkylphenols (APs) in the Yongding River and Beiyun River. The differences in the concentrations and spatial distribution patterns of the nine APs were systematically evaluated using principal component analysis (PCA). The results demonstrated that the APs were widely present in both rivers, and the pollution risks associated with the APs were more severe in the Yongding River than in the Beiyun River. This study provides theoretical data support and a basis for AP pollution risk evaluation in Yongding River and Beiyun River. Triclosan (TCS), a commonly used antibacterial preservative, has been demonstrated to have high toxicological potential, and it adversely affects water bodies [3]. Lu et al. (contribution 6) addressed the adverse effects of TCS on freshwater microalgae (E. gracilis), including morphological alterations, reduced photosynthesis, and oxidative stress. They showed that the main toxic mechanisms of TCS exposure for E. gracilis were changes in ROS and antioxidant enzyme activities, which stimulated algal cell damage, and the inhibition of the TCA cycle metabolic system, including carbon metabolism, nitrogen metabolism, and the D-glutamine and D-glutamate metabolism pathways controlled by the downregulation of DEGs, which were further manifested as oxidative stress and photosynthesis inhibition effects. Wang et al. (contribution 7) investigated the Yellow River Estuary region and found that a total of 34 antibiotics, including macrolides, sulfonamides, quinolones, tetracyclines, and chloramphenicol, were pollutants. The results show that antibiotics were widely present in the water bodies of the Yellow River Estuary, with 14 antibiotics detected to varying degrees, including a high detection rate for lincomycin hydrochloride. Farming wastewater and domestic sewage were the primary sources of antibiotics in the Yellow River Estuary. This study provides beneficial information for the assessment of the ecological risk presented by antibiotics in the Yellow River Estuary water bodies and a scientific basis for future antibiotic pollution control in the Yellow River Basin. Hou et al. (contribution 9) conducted a comprehensive field investigation of Baiyangdian Lake and assessed the ecological risk of PAEs, which can provide data support and a theoretical basis for the formulation of water quality standards and the future prevention and control of PAE pollution. The Water Quality Criteria (WQC) for the protection of aquatic organisms mainly focus on the maximum threshold for pollutants that do not have harmful effects on aquatic organisms. Feng et al. (contribution 13) systematically discussed an overview of water biological conservation, its theoretical methods, and its research progress and detailed the key scientific issues that need to be considered in WQC research. Combined with the specific characteristics of emerging pollutants, some new ideas and directions for future research on the WQC protection of aquatic organisms were proposed. Liu et al. (contribution 12) comprehensively reviewed the development process of WQC in China, focusing on the methodological progress and challenges in selecting priority pollutants, biological screening tests, and standardizing ecotoxicity testing protocols. They also provided critical assessments of the necessary minimum data requirements for quality assurance measures, data validation techniques, and ALC assessment. Moreover, in Men et al.’s (contribution 8) study, a non-experimental approach was used to calculate the RfD values, which explored the potential correlation between toxicity and physicochemical characteristics and the chemical structure of pesticides. The molecular descriptors of contaminants were calculated using T.E.S.T software from the EPA, and a prediction model was developed using a stepwise multiple linear regression (MLR) approach. Approximately 95% and 85% of the data points differed by less than ten-fold and five-fold between the predicted values and true values, respectively, which improved the efficiency of the RfD calculation. The model prediction values have certain reference values in the absence of experimental data, which is beneficial to the advancement of contaminant health risk assessment.

## 3. Conclusions

The papers published in this Special Issue include investigations and the risk assessments of organic pollutants in multiple regions, providing a scientific basis for governments and businesses to formulate environmental policies and technological improvements. The toxicity mechanism research provides a foundation and reference for subsequent research. The review paper systematically summarizes epidemiological investigation findings and delves into the mechanism of correlation between exposure to emerging pollutants and blood glucose imbalance. It comprehensively reviews the development process of WQC in China and systematically provides an overview of WQC, its theoretical methods, and research progress for aquatic organism protection. This Special Issue provides important references for subsequent research.
